# Maize Tortillas Fortified with Ayocote and Quintonil Flours: Nutritional and Functional Properties

**DOI:** 10.3390/foods15010021

**Published:** 2025-12-22

**Authors:** Edwin Rojo-Gutiérrez, Leticia Xochitl López-Martínez, Hilda Karina Sáenz-Hidalgo, Juan Manuel Tirado-Gallegos, Hugo Sergio García-Galindo, Ramiro Baeza-Jiménez

**Affiliations:** 1 Laboratorio de Biotecnología y Bioingeniería, Centro de Investigación en Alimentación y Desarrollo, A.C. Av. Cuarta Sur 3820, Fracc. Vencedores del Desierto, Delicias 33089, Chihuahua, Mexico; 2 Laboratorio de Antioxidantes y Alimentos Funcionales, SECIHTI—Centro de Investigación en Alimentación y Desarrollo, A.C. Carr. Gustavo Enrique Astiazarán Rosas 46, Col. La Victoria, Hermosillo 83304, Sonora, Mexico; 3 Facultad de Zootecnia y Ecología, Universidad Autónoma de Chihuahua, Periférico Francisco R. Almada Km 1, Chihuahua 31453, Chihuahua, Mexico; 4 UNIDA. Tecnológico Nacional de México-Instituto Tecnológico de Veracruz. M.A. de Quevedo 2779, Col. Formando Hogar, Veracruz 91897, Veracruz, Mexico

**Keywords:** underutilized species, antioxidant activity, bioactive compounds, staple food

## Abstract

In recent years, there has been a growing interest in developing sustainable food products that consider not only economic but also environmental and social impacts. Therefore, this study aimed to prepare fortified tortillas with ayocote (*Phaseolus coccineus*) and quintonil (*Amaranthus hybridus*) flours, analyzing the nutritional, functional, physical (color, rollability) and textural properties. Different concentration levels (3, 6, and 9%) and flour mixtures (1:1, 2:1, and 1:2) of quintonil/ayocote were evaluated. The most remarkable chemical composition changes were observed for a 9% substitution, where the contents of protein, ash, and fiber were 1.29, 1.79, and 2.73 times greater than those of the control sample, respectively. In the fortified tortillas, both total phenolic content and antioxidant activity decreased. This can be attributed to the distinct chemical interactions present in the prepared mixtures, which restrict the release of bioactive compounds. Changes were also observed in textural and color properties; however, rollability was not compromised. It is worth noting the potential of this innovation, which can contribute to enhancing nutrition and ensuring food safety, promoting the consumption and preservation of the underutilized plant species employed.

## 1. Introduction

Regarding nutrition and food safety, current challenges include developing or improving foods to ensure food safety and a better nutritional status of society. While the low-income population are commonly susceptible to micronutrient and protein deficiencies, the prevalence of obesity, hypertension, diabetes, and cardiovascular diseases are growing in the rest of the population [1,2]. These diseases stem from various issues, including poverty, crop diversity, excessive consumption of ultra-processed foods, and poor dietary habits [3,4]. However, it has also been observed that there is an increasing awareness among people of improving their dietary habits and consuming health-promoting products, resulting in a market demand for novel food products that offer nutritional and positive health benefits. In that matter, functional and fortified foods rich in antioxidants, dietary fiber, and micronutrients, with low fat content, are gaining popularity, due to their correlation with preventing cardiovascular disease and improving the quality of nutrition in the diet [5].

Nowadays, underutilized plants have become more relevant to the food industry due to their potential use as an alternative for the preparation of functional and fortified foods. The use of these plants could minimize food insecurity, revalorize underutilized crops, and significantly contribute to people’s nutrition, health, and income generation, but many of them are still grown wildly and barely studied, limiting their nutritional and economic potentials [6,7,8]. Ayocote bean (*Phaseolus coccineus*) and quintonil (*Amaranthus hybridus*) are some examples of plant species native to Mexico that have not been widely exploited [9,10]. They hold enormous potential as ingredients to produce novel products or improve the nutritional quality of foods, since they are a rich source of phytochemicals (anthocyanins, flavonoids, and phenolic acids) and nutrients, such as protein (2.7–44%), fiber (5.4–22.7%), carbohydrates (53–60%), and minerals (3.1–13%) [11,12]. The fortification of staple foods is considered one of the most effective approaches for enhancing the nutritional quality of the population’s diet [13]. Maize is one of the major staple foods worldwide and a traditional food of great relevance in Mexico, as it is part of the population’s basic diet, with a per capita consumption of 75 kg/year. Tortillas are one of the main food products and have become a substantial source of nutrients, contributing approximately 70% and 50% of consumers’ daily calories and protein intake, respectively [14]. Tortillas’ high popularity and versatility make them a potential vehicle for delivering bioactive and nutritional ingredients of interest, which may not only aid in the fight against hunger and malnutrition but also enhance public health. Some studies have evaluated the use of ayocote bean and quelites for the fortification of tortillas. Ayocote bean has been successfully employed for the fortification of biscuits [15], bread [16], and fermented white maize [17], whereas for tortillas, only a single study was found. Sánchez-Villa et al. [18] used isolated proteins from ayocote beans and huazontle (*Chenopodium berlandieri*) flour (alone or in different combinations) to increase the nutritional quality of nixtamalized tortillas, being able to enhance the protein content of tortillas by up to 37% without affecting their sensorial or textural properties. However, their study did not incorporate the whole ayocote bean matrix, nor did it assess the functional properties derived from the joint addition of ayocote isolated proteins and quelite. In this context, evaluating the interaction between the two complete food matrices represents a novel approach to elucidating their synergistic effects on the nutritional and functional quality of fortified tortillas. The ayocote provides high-quality protein and essential minerals, while quintonil contributes dietary fiber and phenolic compounds with antioxidant potential. Furthermore, as a native and drought-tolerant species, they contribute to the conservation of agrobiodiversity, food security, and sustainable crop diversification in Mexico.

Therefore, the aim of this study was to prepare fortified tortillas with ayocote (*Phaseolus coccineus*) and quintonil (*Amaranthus hybridus*) flours, followed by the evaluation of their nutritional, functional, physical (color, rollability), and textural properties. This study will promote the consumption and preservation of the underutilized plant species employed.

## 2. Materials and Methods

### 2.1. Plant Materials

Nixtamalized maize flour (MACSA^®^ Calidad Suprema, Santa Isabel, Mexico) was purchased from a local tortilleria in Meoqui, Chihuahua, Mexico. The ayocote beans (*Phaseolus coccineus*) were acquired from a local market in Toluca, Estado de Mexico, Mexico, and the quelites were harvested in Delicias, Chihuahua, Mexico. Both ayocote beans and quelites were washed with distilled water, dried at 40 °C for 12 h in an incubator (Felisa^®^ model FE-133D; Jalisco, Mexico), and ground in a conventional coffee grinder (IKA all basic mill, IKAWorks, Inc., Wilmington, NC, USA) until flours were obtained (≤500 μm). Flours were kept in commercial polypropylene bags with an airtight seal (Ziploc^®^; Toluca, Mexico) and stored in a desiccator at room temperature until use.

### 2.2. Chemicals

All the reactants and solvents were of analytical grade. Standards for phenolic compounds and antioxidant activity were obtained from Sigma-Aldrich (St. Louis, MO, USA).

### 2.3. Tortilla Production

The fortified maize tortillas were prepared using different mixtures containing 3, 6, and 9% of combined ayocote–quintonil flour and nixtamalized maize flour (MACSA^®^ calidad suprema), as is depicted in Table 1.

For all the prepared formulations, approximately 30 mL of distilled water was added to obtain a homogeneous masa, which was allowed to stand in a sealed container for 30 min. Secondly, approximately 39.8 g of masa was withdrawn to obtain tortillas of 14 cm in diameter and 1.9 mm thick, using a commercial tortilla press. Pressed masa was placed on a hot metal plate (Tramontina^®^ comal; Carlos Barbosa, Brazil) preheated between 280 and 300 °C, where tortillas were cooked on one side for 30 s and then on the other for 50 s, before being turned over again for another 30 s. After that, tortillas were allowed to reach room temperature and packaged in commercial polypropylene bags with an airtight seal (Ziploc^®^) and stored in a desiccator for subsequent proximal, texture, and functional analysis.

### 2.4. Chemical Composition Analysis

Methods used for the determination of moisture, protein, lipids, ash, and crude fiber were conducted according to AOAC Official Methods 925.09 (Moisture in flour), 984.13 (Protein in animal feed, Kjeldahl method), 920.39 (Fat in animal feed), 923.03 (Ash of flour), and 962.09 (Crude fiber in cereals and feeds), respectively [19]. Carbohydrates were calculated by difference:

%carbohydrates = 100% − (%moisture + %protein + %lipids + %ash + %crude fiber)

### 2.5. Capacity of Rollability

When the prepared tortillas reached room temperature, rollability was performed following the method proposed by Arámbula-Villa et al. [20]. Each tortilla was rolled up around a 2 cm diameter stainless steel rod, and the observed degree of breakage of the length of the tortilla was measured. The degree of breakage was evaluated using a scale from 1 to 5 (1 = 0% of the length of the tortilla; 2 = 1 to 25%; 3 = 26 to 50%; 4 = 51 to 75%; 5 = 76 to 100%).

### 2.6. Textural Analysis of Masa

The textural analysis profile of masa was adapted from the methodology described by Topete-Betancourt et al. [21]. The different obtained masa from formulations were allowed to stand for 30 min and shaped into a squared prism employing a 3D printed plastic mold (3 × 3 × 2 cm). The analysis of each sample was carried out using a TAXTPlus texturometer (Stable Micro Systems, Surrey, UK) fitted with a P/25 aluminum cylinder of 25 mm in diameter. The sample was placed on the texturometer platform and subjected to a double consecutive compression cycle of 40% with respect to the original height at a speed of 2 mm/s. From the curves obtained, hardness, elasticity, adhesiveness, and cohesiveness were estimated.

### 2.7. Textural Analysis of Tortilla

Textural analyses were made according to the methodology described by Topete-Betancourt et al. [21], with some modifications. Ten strips (60 × 10 mm) were cut for each formulation, and their thickness was measured at 10 different points using a micrometer (Mitutoyo, Kobe, Japan). The strips were subjected to tensile stress on a TAXTPlus texturometer (Stable Micro Systems, Surrey, UK) with a 30 kg load cell. The tensile tests were carried out at a strain rate of 2 mm/s and a distance between the chucks of 4 cm. The stress at fracture [22] in MPa, the percentage of deformation (%E), and the modulus of elasticity (ME) in MPa were determined.

### 2.8. Color Measurement

The color of tortillas was measured using a portable Konica Minolta DP-400 colorimeter (Minolta Co., Ltd., Osaka, Japan), in accordance with the method of Páramo-Calderón et al. [23]. Readings were established at three different points on the tortillas’ surface, and they were performed in triplicate. Color coordinates were obtained in terms of the CIELAB (Commission Internationale de I’Eclairage) system, where luminosity (L*) was considered as the range from white to black, a* as the range from red (positive values) to green (negative values) tones, and b* as the range from yellow (positive values) to blue (negative values) tones. a* and b* parameter values were used to calculate hue angle (h*) and chroma (C*).

### 2.9. Obtention of the Extracts

Extraction samples were obtained by ultrasound-assisted extraction using a 1:5 tortilla/solvent ratio (0.25 g/5 mL) and an hydroethanolic mixture (25% distilled water, 75% ethanol) as the solvent [24]. Samples were collected in amber vials containing the hydroethanolic solution and subsequently sonicated in a water bath (Sonicator VWR model 150 D; VWR International., West Chester, PA, USA), for 30 min at 45 °C. The resulting mixtures were filtered through filter paper #2 (Whatman, Maidstone, UK) and separated by centrifugation (Eppendorf centrifuge 5804R, Poway, CA, USA) at 8000 rpm for 15 min. Supernatants were recovered and stored in amber vial tubes at 4 °C until use.

### 2.10. Determination of the Total Phenolic Content (TPC)

TPC was determined according to the method described by López-Martínez et al. [24]. Sample aliquots (20 μL) were combined with 20 μL of Folin–Ciocalteu reagent and incubated for 5 min. Then, 20 μL of 0.01 M Na_2_CO_3_ were added and the resulting mixture was incubated for another 5 min. Finally, 125 μL of distilled water were added to the mixture and the absorbance was measured at 790 nm in a microplate reader (Multiskan Go, Thermo Scientific, Vantaa, Finland). A standard calibration curve was prepared using gallic acid (0–6 mg/mL), and the amount of TPC in each extract was calculated and expressed as mg of gallic acid equivalent per mg of dried sample (mg GAE/mg ds).

### 2.11. Antioxidant Capacity Assays

The evaluation of antioxidant activity was measured using DPPH and ABTS assays, in accordance with the methodologies described by López-Martínez et al. [24]. DPPH was performed by reacting 7 μL of the sample extract with 193 μL of DPPH solution. The mixture was incubated in the dark for 30 min at room temperature, and the decrease in absorbance at 517 nm was measured using a microplate reader (Multiskan Go, Thermo Scientific, Vantaa, Finland). A standard calibration curve was prepared using Trolox (0–0.4 µmol/mL) to express the results as mg of Trolox equivalents per milliliter (mg TE/mL). For ABTS, 10 μL of the sample extract were allowed to react with 190 µL of ABTS radical. The mixture was incubated for 1 min and afterwards the absorbance was measured at 734 nm in a microplate reader (Multiskan Go, Thermo Scientific, Vantaa, Finland). A standard calibration curve was prepared using Trolox (0–0.7 µmol/mL) to express the results as mg of Trolox equivalents per milliliter (mg TE/mL).

### 2.12. In Vitro Gastrointestinal Digestion and Phenolic Bioaccessibility Index

The digestive process was simulated to measure the bioaccessibility of phenolic compounds present in the fortified tortillas, adapted from Andrade et al. [25]. Briefly, the digestion was initiated with the oral phase by adding 2 mL of artificial saliva (oral phase) to 3 mL of tortilla extracts in digestion tubes, followed by stirring (200 rpm, 2 min, 37 °C, pH 6.0 ± 0.02). The resultant mixture was combined with 4 mL of gastric juice and incubated (130 rpm, 120 min, 37 °C, pH 2.0 ± 0.02) for the gastric phase. After that, 4 mL of intestinal juice was added and incubated (45 rpm, 120 min, 37 °C, pH 6.0 ± 0.02) for the intestinal phase. The digestion tubes were centrifuged at 10,000 rpm for 10 min at 4 °C at the last phase of the in vitro digestion, and the resultant supernatant was stored at −20 °C for subsequent analysis. As controls, samples without tortilla extracts were employed. Each sample was processed by triplicate for a total of 30 separate digestions. The bioaccessibility index was calculated as the percentage of total phenolic content following the methodology reported by Sánchez-Rodríguez et al. [26].

### 2.13. Shelf-Life

Microbiological (molds and yeasts), moisture, and rollability analysis were conducted on the fortified tortillas. Thus, two groups were submitted to that analysis: one was kept at ~32 °C and the other at ~4 °C, monitored at 0, 3, and 7 days. Molds and yeasts were analyzed under the protocols described by the Official Mexican Standards NOM-187-SSA1/SCFI-200 [27] and NOM-111-SSA1-1994 [28], respectively.

### 2.14. Statistical Analysis

All experiments were analyzed using a completely randomized design. For all the experimental analyses (except colorimetry), a one-way analysis of variance and Tukey’s test (*p* ≤ 0.05) were carried out. Since the color values of tortillas were not normally distributed, a Kruskal–Wallis test was performed to determine statistically significant differences.

## 3. Results and Discussion

### 3.1. Chemical Composition

The nutritional composition of the different fortified tortillas can be observed in Table 2. The percentage of fortification (3, 6, and 9%) of tortillas, as well as the ratio of ayocote (AF) and quintonil (QF) flours employed, significantly modified the chemical composition of the tortillas obtained. The protein, ash, and fiber contents increased as the percentage of fortification did. Conversely, the carbohydrate content and the caloric intake significantly decreased. Moisture content, on the other hand, increased or decreased depending on the level of fortification and ratio of AF:QF utilized. The tortillas containing an even (T3F1) or major (T3F3) AF ratio at a 9% fortification level exhibited the highest protein content, registering increases of 16.9% and 29.4%, respectively, compared to the control tortilla. These results are in accordance with those reported by Sánchez-Villa et al. [18], who were able to increase the protein content of maize tortilla by up to 30% when adding 10% of *P. coccineus* protein concentrate. It is worth noting that they achieved such increment by substituting 1% more of maize flour and employing a protein concentrate. Therefore, the same results were obtained by supplementing with 9% AF and QF in a 2:1 ratio. The present study achieved higher result values in protein content compared to the findings reported by Treviño-Mejía et al. [29], who reported an increment of 15.4% in protein content by incorporating 20% of common bean flour. On the other hand, the results attained in this work were lower than the reported data of Salazar et al. [30], who used faba (*Vicia faba*) and white (*Phaseolus vulgaris*) bean flours to supplement maize tortillas. They obtained a protein increase of 98% and 132%, respectively. However, they reported a 75% partial substitution of maize flour for both beans used to fortify maize tortillas.

The ash content significantly increased when a 6 and 9% of AF and QF were added. However, the highest values of ash content were presented by the treatments with major QF ratios, being able to increase the ash content by 77.6 and 79% (T2F2 and T3F2, respectively), compared to their control sample. These results are in accordance with Sánchez-Villa et al. [18], who also observed that the ash content of their fortified tortillas increased as the concentration of huazontle increased. When they added 10% of quelite, the ash content increased by 94%. But the treatment with 2.5% of *P. coccineus* and 7.5% of huazontle exhibited an increased ash content of 78.5%, which is very similar to the value obtained by the treatment T3F2 of the current study, which contains 6% of quelite. It is also worth noting that Salazar et al. [30], despite adding 25% white bean flour, reported a much lower increase value of 35.7% in ash content in comparison to their control sample; however, when they added 25% of white bean flour, the registered increase in content was 85.7%, which is only slightly higher, considering that they used 14% more plant material to fortify their maize tortilla.

Crude fiber was significantly enhanced by all the fortification levels, but just like ash content, the highest value was observed at a fortification level of 9%, by treatment T3F2, which is constituted of a high QF ratio, which is a rich source of crude fiber and minerals. This increase value was higher than the results obtained by Sánchez-Villa et al. [18], who did not observe significative modifications in fiber content, and Salazar et al. [30], who registered an increase of 169.5% in fiber content when 75% of white bean was added. The moisture content of tortillas ranged between 49.5 and 61.42%. It was observed that when the concentration of AF predominated over the QF in the tortilla formulations, the moisture tended to decrease in comparison to the control tortilla. The lower moisture values (*p* > 0.05 between them) were exhibited by treatments T1F3 (49.5%) and T3F3 (49.67%), which are approximately 9% lower than that of the control tortilla. Moisture content was only statistically significantly increased (13.2% in comparison to the control tortilla) when a high QF concentration was employed at the higher level of fortification (9%, T3F2), due to the ability of quintonil to absorb water. This ability can be attributed to the fact that quintonil has a high fiber content. A higher moisture level is required to correctly hydrate the masa and achieve the desired consistency for tortillas with a higher fiber content [18]. This agrees with other studies where tortillas were fortified with ingredients rich in fiber content [18,31,32]. It is worth noting that treatment T3F2 also significantly decreased the caloric intake, by 24.46%. Only treatment T3F3 showed a significant effect on the lipid content of tortillas, leading to a decrease of 27.4% in comparison to the registered value of the control tortilla. Ayocote contains a low lipid concentration, so it probably diluted the lipid content of tortillas.

The observed increase in protein, ash, and fiber content of the fortified tortillas could have an impact on population diets by helping to meet the nutritional recommended intakes of the nutrients, especially where tortillas are highly consumed (as in Mexico).

### 3.2. Tortilla Color

Color measurements of tortillas revealed significant modifications when plant flours were incorporated (Table 3). The use of QF resulted in tortillas exhibiting a greener hue that could be visually observed. Green coloration appeared to intensify as the concentration of QF increased. Color modification was mainly attributed to the use of QF, as the ayocote bean is considered a white bean variety, whereas QF has a dark green color. These observations were confirmed with the results obtained from the L* and a* parameters. Luminosity (*L*) decreased from 79 to 54.8 (T3F2), while h* value increased by up to 5 degrees (96.29, T3F2) compared to the control sample. Therefore, supplemented tortillas became greener and less luminous as the fortification level and QF ratio increased. Moreover, treatments T2F1 and T3F2 showed a significantly higher croma (C*) value in comparison to the rest of the treatments (as well as the control), supporting the supposition that the alteration in the coloration of tortillas is attributed to the inclusion of QF. These results are in accordance with Páramo-Calderón et al. [23], who used *Moringa oleífera* flour to fortify maize tortillas. Like QF, moringa flour also presents a naturally dark green color.

### 3.3. Textural Properties of Masa and Tortilla

Table 4 shows the texture parameters of maize masa. The addition of quintonil and ayocote resulted in maize doughs with significantly less hardness. T3F2 presented the lowest value, indicating that it was the most deformable masa sample. It has been observed that tortillas with higher fiber content tend to exhibit lower hardness values. Fiber competes for water and disrupts starch gelatinization during hydration, which limits the interactions between protein and other components, resulting in softer and less compact structures. Consequently, the overall structure of the tortilla becomes more brittle [33]. It should also be considered that the increased water-binding capacity of the fiber may also contribute to a more hydrated and deformable masa, explaining the lower hardness values observed. The degree to which the sample sticks to your teeth during mastication is known as adhesiveness. In other words, it is the effort required to overcome the attraction force between food and other surfaces [34]. Only an incorporation of 9% of AF and QF (1:2, T3F2) produced a significant difference in the adhesiveness of the masa. These results suggest the predominance of quintonil resulted in higher adhesiveness values. This may be attributed to the presence of soluble fiber and phenolic compounds, which are capable of interacting with starch and proteins through hydrogen bonding, thereby increasing the stickiness of the hydrated dough [35]. Suitable adhesiveness enables the dough to adhere to the rolling mills of the tortilla machine, forming dough discs, and then properly separate from them during the cooking process, allowing for good machinability [36]. The presence of negative adhesiveness values indicates that the stress is oriented in a downward direction and could be related to a very high adhesiveness. However, all obtained values are over −0.5 N·s, except for the value obtained by treatment T3F2, which is still significantly higher than other reported values of adhesiveness (≤−3.94 N) by other studies [37,38]. Experimentally, all masa treatments could be easily molded into conventional discs using a manual press and removed from it without difficulty, allowing for the cooking step to proceed. As with adhesiveness, cohesiveness, commonly defined as the strength of the internal bonds making up the body of the product, highly affects the machinability of the dough and tortillas. Masa with cohesive properties allows it to support external forces, such as mixing, without becoming brittle [21], and generates a curtain on the sheeting and forming rollers, facilitating the detachment of the cut tortilla discs from the rollers [39]. Cohesiveness was found only to be affected when 9% AF and QF (1:2) was incorporated (T3F3), increasing (*p* ≤ 0.05) the cohesiveness of the dough by 63.4% (compared to the control sample). This increase can be associated with its protein content, since it enhances the interaction of starch–protein–lipid components [39]. Additionally, ayocote globulins may participate in calcium-mediated crosslinking (resulting from nixtamalization), further strengthening the internal structure of the masa [40,41]. Similar results were reported by Hernandez-Chavez et al. [14], who added 2.5, 5, and 7% of lupin flour into maize tortillas. They found that the increase in the cohesiveness of the tortillas was related to the protein content of Lupinus, and registered cohesiveness values ranged between 0.117 and 0.130.

The treatments derived from a 9% fortification showed the most significant differences compared to the control, where the T3F3 treatment differed in the hardness and adhesiveness parameters. In contrast, the T3F2 treatment exhibited differences in all parameters. A fortification of less than 9% showed no changes in the texture of the dough, except for hardness. On the other hand, the texture of the tortilla was also affected due to the incorporation of quelite and ayocote. Treatments T1F1, T2F2, and T3F2 required the same force for breaking, which was lower than that recorded by the control. Treatment T3F3 was the only one that required significantly greater force for rupture. In terms of elongation, there was no statistical difference between the treatments; however, a comparison with the control revealed a lower elongation distance for the latter.

The textural parameters of tortillas were also affected (*p* ≤ 0.05), using plant flours for their fortification (see Table 5). It was observed that treatments T1F1, T2F1, and T3F2 exhibited a lower tensile strength value compared to the control tortilla. As shown in Table 2, the addition of AF and QF generally increased the fiber content in all treatments. Higher fiber contents typically produce softer tortillas, due to a reduction in the amount of gelatinized starch. The physical presence of particles interferes with starch retrogradation, limiting the formation of a continuous crystalline network and producing more flexible but mechanically weaker tortillas [33].

On the other hand, treatment T3F3 exhibited the highest tensile strength. This could be related to the AF and its level concentrations contained in the tortilla. The additional legume proteins could act as structuring agents within the tortilla matrix, enhancing firmness and resistance to rupture. Similar strengthening effects have been reported in tortillas enriched with white (*P. vulgaris*) and faba bean (*V. faba*) flours [30]. Moreover, it is known that beans contain solid components such as polysaccharides and non-cellulosic compounds, which can heavily influence the functional properties of food. These properties include thickening, stabilization, emulsification, gelation, and film formation [42]. The presence of these polysaccharides in the AF could be impacting the hardness of the produced tortillas. All tensile strength values were lower than those reported by Páramo-Calderón et al. [23], who worked with *M. oleífera* and obtained values ranging from 6.79 to 9.38 N. However, the tensile strength from the control tortilla of the present study is slightly lower than the bottom range value (1.52 N) of nixtamalized corn tortillas reported by Grijalva et al. [43] and higher than that reported by Treviño-Mejía et al. [29] of a commercial nixtamalized tortilla (1.03 N). With respect to extensibility, all treatments exhibited higher values (*p* ≤ 0.05) than the control sample, but without any difference between them. With respect to rollability, all treatments were demonstrated to be just as flexible as the control tortilla, without rupture (see Table 5).

### 3.4. Total Phenolic Content and Antioxidant Activity Through Simulated Gastrointestinal Digestion

The total phenolic content (TPC) and antioxidant activities of undigested and digested extracts samples are shown in Table 6. The control tortilla exhibited a TPC of 1.73 mg GAE/g (dw). The TPC of undigested tortilla extracts ranged from 0.31 to 0.96 mg GAE/g (dw), whereas that of digested samples ranged from 0.21 to 1.43 mg GAE/g (dw). Therefore, the partial substitution of maize resulted in a decrease in the TPC of formulated tortillas. The latter could be attributed to a dilution effect by the addition of AF, since preliminary analysis showed that tortillas fortified only with AF (9%) presented lower TPC (1.22 ± 0.22 mg GAE/g [dw]) than quintonil (9%, 1.74 mg GAE/g [dw]) and maize tortillas. Another reason could be attributed to antagonistic interactions among polyphenols. These behaviors have also been documented in other tortilla fortification studies. Fortified tortillas with extruded amaranth flour exhibited reduced antioxidant activity compared to pure blue maize tortillas, even though their overall nutritional quality improved [44]. The authors attributed this reduction to the lower phenolic content of the added amaranth ingredient and to potential inhibitory interactions among phenolic structures. Similar behavior was observed in sorghum-bran-fortified tortillas, where phenolics strongly associated with insoluble dietary fiber limited their extractability and reduced the measured antioxidant activity [45].

Significant changes (*p* < 0.05) in the TPC were observed at all stages of in vitro digestion, with tortillas formulated with 9% plant fortification presenting the highest concentrations. During the oral and intestinal stage, the TPC of treatments T1F1 and T2F1 decreased, whereas during the gastric digestion phase (GDP) no significant changes were registered. For treatments T3F2 and T3F3, a decrease in their TPC was also observed during the oral digestion phase (ODP), but during the GDP, their TPC increased by 1.76 and 1.68 times (T3F2 and T3F3, respectively). No significant changes were observed in their TPC during the intestinal digestion phase (IDP) [46]. Overall, lower values (*p* < 0.05) of TPC were obtained from all samples after the ODP in comparison to undigested samples (see Table 6). Subsequently, the TPC significantly increased in all treatments (GDP). TPC increased 19-fold for treatment T1F1 and approximately 4-fold for treatments T2F1, T3F2, and T3F3. T1F1 and T2F1 did not show significant differences in TPC values compared to their non-digested samples, whereas the contents in T3F2 and T3F3 were still higher than in their undigested values (1.71 and 1.5 times higher, respectively). The increase in TPC during the GDP is attributed to enzyme effects and the acidic conditions that prevail in this stage, which hydrolyze food matrices and release bound phenolic compounds. Moreover, the stability of phenolic compounds is enhanced in such environment, avoiding the loss of phenolic compounds (PCs) [24,47]. Conversely, a decreasing trend of TPC was observed after the IDP. Nevertheless, treatments did not show significant TPC changes in reference to their non-digested and ODP values, except for T1F1, which remained 10.5 times higher than the latter. Just as pH/enzymes affect the GDP during the release of PCs by disrupting food matrices, as previously explained, these factors can potentially alter the phenolic hydroxyl group as well. This modification can affect the antioxidant activity of the released phenolics, leading to either a decrease or an increase in their content and activity in the IDP [47]. This behavior of the TPC during the stages of gastrointestinal digestion has been observed in other studies, where different plant materials, such as huitlacoche and various oregano species and fruit seeds, have been analyzed [24,48,49]. On the other hand, these results partially contradict those of Menchaca-Armenta et al. [50], who reported that the TPC of an extruded nixtamalized blue tortilla and traditional nixtamalized white tortilla increased after every digestion phase. Still, the higher solubilization rate of PCs was observed in the IDP (intestinal > gastric > oral), whereas in the present work, it was in the GDP (gastric > intestinal > oral). This trend has been reported for other fortified maize systems. Acidic gastric conditions and pepsin activity promote the release of bound phenolic acids and flavonoids from the tortilla matrix, as documented in brown seaweed-fortified tortillas [51] and in amaranth–maize tortillas enriched with extruded flours [44]. The decline of TPC values from maize-based matrices during the IDP is generally attributed to the structural transformation, degradation, or reduced solubility of phenolics under near-neutral pH conditions [51].

The bioaccessibility index (BI) values of TPC obtained after in vitro gastrointestinal digestion of the fortified tortillas prepared are shown in Table 7. The absorption of phenolic compounds is influenced by their chemical structure and how they interact with the food matrix. The BI values among all treatments varied significantly: T3F2 > T3F3 > T1F1 > T2F1. Low BI may result from a block of α-amylase activities due to the interaction of PCs with nutrients such as fiber, starch, or proteins, avoiding the bind of the PCs to the active site of the enzyme and forming insoluble complexes, limiting the release of bound PCs from the food matrix into the soluble fraction [52]. The BI values calculated also reveal the effect of the previous gastric phase, where the acidic environment softens and hydrates dietary fiber while simultaneously unfolding and partially hydrolyzing proteins. These structural changes open the food matrix, allowing digestive fluids to access and release previously bound phenolic compounds, thereby increasing bioaccessibility [53]. Despite PCs usually being absorbed in the intestines, they also can be absorbed in the stomach [54,55].

With respect to antioxidant activity, the control sample exhibited the highest DPPH and ABTS scavenging values. It is well-known that antioxidant activity is commonly correlated with the content of phenolic compounds [56]. However, it is also important to consider that the antioxidant capacity of PCs depends on its chemical structure and interactions between these chemicals [57]. Therefore, the dilution effect of TPC observed in the formulated tortillas and their antagonistic interactions could be the main reason that treatments exhibited lower scavenging capacities than the control tortilla. In general, the DPPH and ABTS scavenging capacity followed the same behavior as the TPC changes during the simulated gastrointestinal digestion. In this regard, the highest antioxidant values of DPPH were observed at the end of the GDP and corresponded to treatments T3F3 and T3F2 (6.21 and 6.64 mg TE/g dw, respectively). Hence, the DPPH scavenging capacity of T3F2 significantly increased by 16.3% compared to its undigested sample (5.71 mg TE/g dw). T3F3, on the other hand, did not present a significant difference in comparison to its undigested value (6.91 mg TE/g dw).

Regarding the ABTS assay, as mentioned earlier, like TPC and DPPH, the highest ABTS scavenging values were obtained at the end of GDP, again by treatments T3F2 and T3F3 (3.61 and 4.08 mg TE/g dw). However, unlike DPPH, the antioxidant values of both treatments increased significantly by approximately 1.3 times when compared to their undigested sample values. Overall, the DPPH and ABTS scavenging capacity of the treatments did not show significant differences between the ODP and IDP, except for T3F2 and T3F3, which increased their DPPH and ABTS scavenging capacity by (*p* < 0.05) 1.3 times, respectively, at the end of the IDP.

The phenolic classes likely responsible for their antioxidant properties have been characterized in studies of maize, legumes, and amaranth species. The phenolic compounds most likely driving the trends observed in this study correspond to well-characterized subclasses reported in maize, legumes, and quelite species. Nixtamalized maize is rich in hydroxycinnamic acids (primarily ferulic and p-coumaric acids) and flavonoids such as quercetin, rutin, nicotiflorin, and isoquercitrin, with anthocyanins present in pigmented varieties [58]. Ayocote bean flour contains a diverse profile of phenolic acids, including gallic, chlorogenic, vanillic, and caffeic acids, as well as flavonoids such as rutin and quercetin derivatives [59]. Similarly, quintonil provides ferulic and chlorogenic acids, as well as flavonoids such as rutin and phloridzin [60]. These compounds contribute to antioxidant, antihypertensive, and hypoglycemic activities traditionally associated with quelites. Previous functional tortilla studies have shown that these phenolic subclasses are highly responsive to processing and digestion, with gastric acidity promoting their release from fiber–protein–starch complexes [44,61]. Thus, the dominance of hydroxycinnamic acids and flavonoids across maize, ayocote, and quintonil likely explains the sharp increases in TPC and antioxidant activity during the gastric phase and the subsequent decline under near-neutral intestinal conditions.

### 3.5. Evaluation of Shelf-Life of the Fortified Tortillas

The shelf life of tortillas is typically limited by their high moisture content and water activity. Because of that, tortillas are preferably consumed freshly made, so that consumers can enjoy the highest quality of their sensorial attributes. Table 8 shows the modifications observed in the tortillas over time (7 days) in terms of moisture, rollability, and growth of molds and yeasts. The latter is one of the leading causes that limits the preservation of tortillas, since their growth can only be delayed but not avoided [62]. As shown in Table 8, the moisture content of treatments T2F1, T3F2, and T3F3 was significantly altered (*p* < 0.05) when tortillas were stored in a refrigerator at 4 °C. The moisture content of treatment T2F1 and T3F2 decreased, while that of treatment T3F3 increased. These moisture variations could be related to the water exchange between tortillas and their environment, which is influenced by the different moisture levels within the tortillas and the relative humidity present in the fridge. It is known that the physical properties of stored vegetables can be influenced by the relative humidity levels, such as weight (gain or loss of moisture) and color [63].

As expected, the storage time impacted the flexibility of tortillas. Rollability overall tended to decrease as time elapsed, being more affected by the tortillas stored at a cooler temperature (4 °C). Tortillas become more brittle over time due to the retrogradation of amylose and amylopectin, which is one of the primary causes of their deterioration [64]. In addition, Campas-Baypoli et al. [65] reported that maximum loss of flexibility of tortillas occurs when they are stored at refrigeration temperatures (3–10 °C), due to the enhanced rate of retrogradation of amylopectin. Treatments stored at room temperature for 3 days or more (except for T2F1) exceeded the permissible levels (300 UFC/g) established by the Official Mexican Standard NOM-111-SSA1-1994 for mold and yeast counts.

On the other hand, all refrigerated stored tortillas remained suitable for consumption, as they showed no presence of molds and yeasts throughout the 7-day storage period, which is a significant value considering that no additives were used in their formulations. The fortified tortillas prepared in the present work showed a longer shelf life (refrigeration temperature) in comparison to the results reported by Heredia et al. [66], who used freeze-dried jumbo squid muscle flour to supplement maize tortillas (2.5 and 5%, *w*/*w*). The molds and yeasts evaluation at days 3 and 5 of shelf life established that their supplemented tortillas could be stored for up to 3 days only, since at day 5 the molds and yeasts levels did not comply with the respective standards.

## 4. Conclusions

The fortification of maize tortillas with ayocote and quintonil flours resulted in products with enhanced nutritional attributes while maintaining adequate technological characteristics. The 9% incorporation level provided the most favorable outcome, significantly increasing protein, fiber, and mineral content without negatively affecting color or texture. Textural responses varied according to the dominant ingredient: quintonil reduced hardness and tensile strength due to its higher fiber content, whereas ayocote increased cohesiveness and mechanical resistance because of its greater protein contribution. During in vitro digestion, fortified tortillas exhibited lower TPC in the undigested state but showed marked increases in the gastric phase, suggesting an improved release of bound phenolic compounds under acidic conditions.

Overall, the findings highlight the potential of incorporating underutilized native species to produce nutritionally improved tortillas without compromising product quality. Future research could consider LC–MS characterization of phenolic profile and the evaluation of bioactive stability over storage.

## Figures and Tables

**Table 1:** Formulation of the different fortified flours for tortilla production.

Formulation *	Quintonil/Ayocote Ratio	Treatment	Nixtamalized Maize Flour (g)	Quintonil Flour (g)	Ayocote Flour (g)
0%	-	Control	16	-	-
3%	1:1	T1F1	15.52	0.24	0.24
	2:1	T1F2	15.52	0.32	0.16
	1:2	T1F3	15.52	0.16	0.32
6%	1:1	T2F1	15	0.5	0.5
	2:1	T2F2	15	0.67	0.33
	1:2	T2F3	15	0.33	0.67
9%	1:1	T3F1	14.6	0.7	0.7
	2:1	T3F2	14.6	0.94	0.46
	1:2	T3F3	14.6	0.46	0.94

* Fortification percentage with different blends of quintonil and ayocote flours.

**Table 2:** Proximal composition of the different tortilla formulations (%).

Treatments	Moisture **	Protein *	Lipids *	Ash *	CHO’s *	Crude Fiber *	Caloric Intake **
Control	54.28 ± 0.69 ^bc^	8.03 ± 0.03 ^d^	1.46 ± 0.20 ^a^	1.25 ± 0.00 ^e^	89.26 ± 0.12 ^a^	4.81 ± 0.18 ^e^	177.49 ± 3.48 ^abcd^
T1F1	50.47 ± 1.45 ^cd^	8.16 ± 0.04 ^d^	1.47 ± 0.16 ^a^	1.50 ± 0.00 ^de^	88.87 ± 0.14 ^ab^	6.35 ± 0.15 ^de^	190.00 ± 7.14 ^ab^
T1F2	51.29 ± 0.74 ^bcd^	8.13 ± 0.20 ^d^	1.40 ± 0.22 ^ab^	1.52 ± 0.18 ^de^	88.95 ± 0.43 ^ab^	6.92 ± 1.04 ^bd^	185.54 ± 2.00 ^abcd^
T1F3	49.50 ± 1.35 ^d^	8.17 ± 0.17 ^d^	1.33 ± 0.24 ^ab^	1.53 ± 0.01 ^de^	88.97 ± 0.32 ^ab^	6.27 ± 0.56 ^de^	193.70 ± 6.90 ^a^
T2F1	55.48 ± 1.29 ^b^	8.65 ± 0.08 ^cd^	1.35 ± 0.24 ^ab^	1.69 ± 0.25 ^bc^	88.31 ± 0.07 ^cd^	8.12 ± 0.70 ^bc^	166.50 ± 6.83 ^d^
T2F2	54.30 ± 1.21 ^bc^	8.22 ± 0.46 ^d^	1.30 ± 0.20 ^ab^	2.22 ± 0.01 ^ab^	88.26 ± 0.26 ^cde^	8.13 ± 0.29 ^bc^	171.09 ± 5.26 ^bcd^
T2F3	50.72 ± 1.45 ^cd^	9.08 ± 0.30 ^d^	1.25 ± 0.11 ^ab^	1.78 ± 0.03 ^cd^	87.89 ± 0.27 ^bc^	6.77 ± 0.59 ^cd^	187.75 ± 6.65 ^abc^
T3F1	54.41 ± 1.50 ^bc^	9.39 ± 0.33 ^bc^	1.24 ± 0.06 ^ab^	2.24 ± 0.06 ^ab^	87.13 ± 0.32 ^ef^	8.45 ± 0.06 ^b^	169.95 ± 7.36 ^cd^
T3F2	61.42 ± 1.08 ^a^	8.65 ± 0.07 ^cd^	1.53 ± 0.08 ^a^	2.45 ± 0.19 ^a^	87.37 ± 0.03 ^def^	13.15 ± 1.56 ^a^	134.07 ± 6.53 ^e^
T3F3	49.67 ± 0.94 ^d^	10.39 ± 0.67 ^a^	1.06 ± 0.04 ^b^	1.81 ± 0.13 ^cd^	86.74 ± 0.43 ^f^	8.33 ± 0.97 ^b^	193.69 ± 10.9 ^a^

Different letters in the same column indicate significant differences (*p* ≤ 0.05). Values are expressed as the mean ± standard deviation (n = 3). * Dry weight ** Fresh weight, caloric intake calculated in kcal/100 g; CHO’s = carbohydrates.

**Table 3:** Effect of the incorporation of quintonil and ayocote flours on the color parameters of tortillas.

Treatment	L*	a*	b*	C*	h*
Control	79.02 ± 0.98 ^a^	−0.42 ± 0.11 ^a^	18.70 ± 0.40 ^b^	18.70 ± 0.40 ^a^	91.30 ± 0.34 ^c^
T1F1	70.59 ± 2.50 ^b^	−1.12 ± 0.04 ^b^	19.01 ± 0.11 ^b^	19.05 ± 0.11 ^a^	93.37 ± 0.12 ^b^
T2F1	62.19 ± 1.25 ^c^	−1.49 ± 0.24 ^c^	19.95 ± 0.08 ^a^	20.01 ± 0.66 ^b^	94.31 ± 0.66 ^b^
T3F2	54.80 ± 1.92 ^c^	−2.20 ± 0.22 ^c^	20.02 ± 0.34 ^a^	20.14 ± 0.33 ^b^	96.29 ± 0.66 ^a^
T3F3	62.65 ± 1.60 ^d^	−1.39 ± 0.20 ^d^	18.54 ± 0.20 ^b^	18.59 ± 0.18 ^a^	94.31 ± 0.66 ^b^

Different letters in the same column indicate significant differences (*p* ≤ 0.05). Values are expressed as the mean ± standard deviation (n = 3).

**Table 4:** Texture profile analysis of masa supplemented with ayocote and quintonil flour.

Treatment	Hardness (N)	Adhesiveness (N·s)	Cohesiveness (N)	Elasticity (mm)
Control	8.45 ± 0.35 ^a^	−0.22 ± 0.09 ^bc^	0.11 ± 0.00 ^b^	0.21 ± 0.01 ^b^
T1F1	7.06 ± 0.28 ^b^	−0.15 ± 0.06 ^c^	0.13 ± 0.01 ^b^	0.23 ± 0.03 ^b^
T2F1	5.87 ± 0.12 ^c^	−0.19 ± 0.00 ^c^	0.12 ± 0.01 ^b^	0.25 ± 0.04 ^b^
T3F2	5.23 ± 0.00 ^d^	−1.70 ± 0.17 ^a^	0.13 ± 0.01 ^b^	0.49 ± 0.13 ^a^
T3F3	5.67 ± 0.15 ^cd^	−0.46 ± 0.05 ^b^	0.18 ± 0.01 ^a^	0.26 ± 0.02 ^b^

Different letters in the same column indicate significant differences (*p* ≤ 0.05). Values are expressed as the mean ± standard deviation (n = 3).

**Table 5:** Textural analysis of fortified tortillas.

Treatment	Tensile Strength (N)	Extensibility (%)	Rollability
Control	1.25 ± 0.04 ^b^	7.58 ± 0.11 ^b^	1
T1F1	1.00 ± 0.08 ^c^	10.43 ± 0.89 ^a^	1
T2F1	0.92 ± 0.03 ^c^	9.79 ± 0.20 ^a^	1
T3F2	1.03 ± 0.05 ^c^	10.86 ± 0.57 ^a^	1
T3F3	2.13 ± 0.14 ^a^	10.19 ± 0.42 ^a^	1

Different letters in the same column indicate significant differences (*p* ≤ 0.05). Values are expressed as the mean ± standard deviation (n = 3).

**Table 6:** Total phenolic compounds and antioxidant activity of in vitro digestion of fortified tortillas extracts.

Digestion Phase	Treatment	TPC (mg GAE/mg ds)	DPPH (mg TE/mL)	ABTS (mg TE/mL)
Undigested	Control	1.73 ± 0.45 ^a^	9.64 ± 0.31 ^a^	5.23 ± 0.31 ^a^
	T1F1	0.31 ± 0.05 ^ghij^	1.75 ± 0.05 ^gh^	0.87 ± 0.01 ^ij^
	T2F1	0.52 ± 0.07 ^efgh^	2.94 ± 0.11 ^ef^	1.96 ± 0.39 ^ef^
	T3F2	0.76 ± 0.08 ^de^	5.71 ± 0.21 ^c^	2.73 ± 0.19 ^d^
	T3F3	0.96 ± 0.11 ^cd^	6.91 ± 0.21 ^b^	3.23 ± 0.15 ^c^
Oral	T1F1	0.02 ± 0.00 ^k^	0.57 ± 0.01 ^i^	0.43 ± 0.02 ^j^
	T2F1	0.14 ± 0.00 ^ij^	1.25 ± 0.01 ^hi^	0.74 ± 0.02 ^ij^
	T3F2	0.34 ± 0.00 ^ghij^	3.09 ± 0.02 ^ef^	1.37 ± 0.23 ^gj^
	T3F3	0.38 ± 0.00 ^fghi^	3.53 ± 0.02 ^de^	1.63 ± 0.07 ^fg^
Gastric	T1F1	0.38 ± 0.01 ^fghi^	1.25 ± 0.03 ^hi^	1.01 ± 0.09 ^hi^
	T2F1	0.55 ± 0.00 ^efgh^	2.56 ± 0.04 ^fg^	1.42 ± 0.01 ^gh^
	T3F2	1.30 ± 0.03 ^bc^	6.64 ± 0.02 ^b^	3.61 ± 0.16 ^c^
	T3F3	1.43 ± 0.01 ^ab^	6.21 ± 0.05 ^bc^	4.08 ± 0.04 ^b^
Intestinal	T1F1	0.21 ± 0.01 ^hij^	0.92 ± 0.04 ^i^	0.81 ± 0.02 ^ij^
	T2F1	0.22 ± 0.00 ^hij^	1.95 ± 0.05 ^gh^	1.04 ± 0.05 ^hi^
	T3F2	0.66 ± 0.00 ^defg^	4.04 ± 0.06 ^d^	1.74 ± 0.16 ^efg^
	T3F3	0.73 ± 0.00 ^def^	4.09 ± 0.05 ^d^	2.17 ± 0.06 ^e^

Different letters in the same column indicate significant differences (*p* ≤ 0.05). Values are expressed as the mean ± standard deviation (n = 3). mg GAE/mg ds: mg of gallic acid equivalent per mg of dried sample. mg TE/mL: mg of Trolox equivalents per milliliter.

**Table 7:** Bioaccessibility index (%) of the fortified tortilla extracts.

Treatment	BI
T1F1	67.74 ± 7.84 ^dB^
T2F1	42.31 ± 11.44 ^cB^
T3F2	86.84 ± 9.13 ^bB^
T3F3	76.04 ± 26.29 ^aB^

Different letters indicate significant differences (*p* ≤ 0.05). Values are expressed as the mean standard deviation (n = 3).

**Table 8:** Shelf-life parameters of fortified tortillas stored at room temperature and 4 °C and room ±temperature for 7 days.

Treatment	T0	D3A	D7A	D3R	D7R
	Moisture (%)				
Control	54.28 ± 0.85 ^a^	54.55 ± 0.70 ^a^	54.22 ± 0.71 ^a^	54.82 ± 0.67 ^a^	54.56 ± 0.97 ^a^
T1F1	50.47 ± 1.78 ^a^	50.47 ± 0.63 ^a^	53.81 ± 1.09 ^a^	49.47 ± 0.62 ^a^	49.00 ± 0.77 ^a^
T2F1	55.48 ± 1.58 ^a^	55.16 ± 0.77 ^a^	55.31 ± 0.52 ^a^	49.08 ± 0.94 ^b^	48.78 ± 0.89 ^b^
T3F2	61.42 ± 1.32 ^a^	61.14 ± 1.06 ^a^	61.03 ± 1.00 ^a^	53.39 ± 1.46 ^b^	52.41 ± 1.00 ^b^
T3F3	49.67 ± 1.15 ^b^	50.03 ± 1.03 ^b^	49.90 ± 1.03 ^a^	53.22 ± 1.02 ^a^	52.92 ± 0.51 ^a^
	Rollability				
Control	1	3	3	4	5
T1F1	1	3	3	4	5
T2F1	1	3	3	4	5
T3F2	1	3	3	4	5
T3F3	1	3	3	4	5
	Yeasts and Molds (UFC/g)				
Control	0	0	0	0	0
T1F1	0	21,000	I	0	0
T2F1	0	0	I	0	0
T3F2	0	18,000	I	0	0
T3F3	0	11,330	I	0	0

Different letters in the same column indicate significant differences (*p* ≤ 0.05). Values are expressed as the mean ± standard deviation (n = 3). D3A = Day 3 stored at room temperature; D7A = Day 7 stored at room temperature; D3R = Day 3 stored at 4 °C; D7R = Day 7 stored at 4 °C. I = Incalculable.

## Data Availability

The original contributions presented in the study are included in the article. Further inquiries can be directed to the corresponding author.
